# A Bayesian modelling framework to quantify multiple sources of spatial variation for disease mapping

**DOI:** 10.1098/rsif.2022.0440

**Published:** 2022-09-21

**Authors:** Sophie A. Lee, Theodoros Economou, Rachel Lowe

**Affiliations:** ^1^ Centre for Mathematical Modelling of Infectious Diseases, London School of Hygiene & Tropical Medicine, London, UK; ^2^ Centre on Climate Change and Planetary Health, London School of Hygiene & Tropical Medicine, London, UK; ^3^ Climate and Atmosphere Research Centre, The Cyprus Institute, Nicosia, Cyprus; ^4^ Barcelona Supercomputing Center (BSC), Barcelona, Spain; ^5^ Catalan Institution for Research and Advanced Studies (ICREA), Barcelona, Spain

**Keywords:** infectious disease dynamics, vector-borne disease, spatial epidemiology, hierarchical modelling, spatial analysis, spatial connectivity

## Abstract

Spatial connectivity is an important consideration when modelling infectious disease data across a geographical region. Connectivity can arise for many reasons, including shared characteristics between regions and human or vector movement. Bayesian hierarchical models include structured random effects to account for spatial connectivity. However, conventional approaches require the spatial structure to be fully defined prior to model fitting. By applying penalized smoothing splines to coordinates, we create two-dimensional smooth surfaces describing the spatial structure of the data while making minimal assumptions about the structure. The result is a non-stationary surface which is setting specific. These surfaces can be incorporated into a hierarchical modelling framework and interpreted similarly to traditional random effects. Through simulation studies, we show that the splines can be applied to any symmetric continuous connectivity measure, including measures of human movement, and that the models can be extended to explore multiple sources of spatial structure in the data. Using Bayesian inference and simulation, the relative contribution of each spatial structure can be computed and used to generate hypotheses about the drivers of disease. These models were found to perform at least as well as existing modelling frameworks, while allowing for future extensions and multiple sources of spatial connectivity.

## 1. Introduction

When modelling infectious disease data across a geographical region, it is important to account for potential spatial connectivity between areas. For example, spatial connectivity may arise from human or vector movement contributing to the spread of a vector-borne disease, or unobservable climatic, behavioural, biological and socio-economic factors shared between areas. Conventionally, Bayesian hierarchical models aim to account for this spatial connectivity by including spatially structured random components within the model [1–3]. Fully Bayesian modelling approaches require the spatial structure of components to be defined prior to model fitting. However, the spatial structure of the data may not be fully known. A recent systematic review found that all Bayesian hierarchical models for mosquito-borne diseases used a distance-based spatial structure, assuming connectivity between regions only exists between neighbours or close observations [4].

Spatial autocorrelation in disease count data may be attributable to multiple sources of connectivity. For example, dengue incidence is associated with climate variation, vector control interventions and levels of immunity in the population which are likely to be shared between close regions [5]. However, dengue is also influenced by human movement which creates links between distant regions that a distance-based spatial connectivity assumption would not capture [6,7]. Long-distance connections are particularly important when studying (re-)emerging diseases which are largely driven by connections between areas experiencing active disease transmission and disease-free areas [8–10]. In these examples, multiple random terms would be required within a Bayesian hierarchical model to capture the different sources of connectivity and quantify the relative importance of each to the disease transmission process.

In this paper, we present a Bayesian hierarchical modelling framework that uses penalized smoothing splines as a flexible method for structuring spatial model components. Smoothing splines use data to inform spatial components, given smoothing assumptions, rather than requiring the full specification of the spatial structure prior to model fitting [11,12]. The result is a non-stationary structure which is setting-specific and requires minimal user assumptions. This approach allows multiple spatially structured random components to be incorporated into the same model and can distinguish between these structures to quantify their relative contribution to the overall spatial structure. Although this study focuses on disease mapping models of count data, we also show that this method can be used for models of binary data.

## 2. Modelling approach

### 2.1. Disease mapping

Disease mapping is an important statistical tool used in epidemiology to explore spatial variation in disease incidence rates. Disease mapping models can generate and test hypotheses about associations between disease and a variety of potential explanatory variables, such as environmental and socio-economic factors [2,13]. Typically, disease counts, *y_i_* (*i* = 1, …, *n*), are collected across a study area separated into *n* contiguous areas. These counts are combined with an offset log(*ξ_i_*) describing the underlying population at risk in each area *i*. For instance, *y_i_*/*ξ_i_* is the empirical incidence rate in *i* when *ξ_i_* is population count. Where a disease is rare or areas within the study are small, estimates of the incidence are highly uncertain and thus unstable and inflated. To overcome this issue, Bayesian (hierarchical) modelling approaches have been developed to allow information from connected regions to be included in the rate estimation using random effects (data pooling). Conventionally, these models take the form

$$y_{i}∼p(E(y_{i}),\psi )$$

and

2.1 $$\log(E(y_{i}))=\log(\xi_{i})+\alpha +S_{i},$$

where *p* is a suitable count distribution (e.g. Poisson, negative binomial), *E*(*y_i_*) is the expected count, *α* is the intercept or baseline risk, *S_i_* are spatially structured random components and *ψ* are hyperparameters of the distribution. The definition of *S_i_* (which describes the spatial structure of *E*(*y_i_*) on the log scale, after correcting for *ξ_i_*) depends on the disease of interest and the assumed spatial structure in the data. A recent systematic review found that spatial statistical models used to study mosquito-borne diseases only considered distance-based connectivity when defining the structure of such spatial random effects [4]. The most common spatial structure assumed connectivity between regions if and only if they share a border using a conditional autoregressive (CAR) model

2.2 $$S_{i}|S_{\;j\neq i}∼N\left(\frac{\sum_{\;j\neq i}W_{ij}S_{j}}{\sum_{\;j\neq i}W_{ij}},\;\frac{\sigma_{s}^{2}}{\sum_{\;j\neq i}W_{ij}}\right)\;,$$

where *W_ij_* are proximity weights, often defined as *W_ij_* = 1 if *i* and *j* share a border, and 0 otherwise. Although the conditional independence assumption intrinsic to neighbourhood-based spatial structures allows for efficient Bayesian computation [14], the nature of spatial connectivity is likely to be more complex and differ across settings. A smooth function with a structure defined using the data rather than prior to model fitting provides a flexible alternative and allows spatial dependency structures to be specific to each setting.

### 2.2. Penalized smoothing splines

Smoothing splines, or smooth functions, are used in generalized additive models to explore nonlinear relationships between a response variable and one or more covariate(s). Smoothing splines are constructed as a linear combination of basis functions, *b_j_* (functions applied to the covariate(s) at given intervals, determined by the type of smoothing spline chosen), multiplied by regression coefficients, *β_j_* [11]. For example,

2.3 $$f(x)=\overset{K}{\underset{\;j=1}{\sum }}\beta_{j}b_{j}(x).$$

Where *f* is a smooth function (the smoothing spline), *x* is the covariate of interest and *K* is the number of ‘knots', or turning points, in the smooth function. The number of knots should be chosen to be large enough that the smooth function adequately describes the data, but not so large that they overfit or become ‘overly wiggly’. To achieve this, a smoothing penalty parameter, *λ*, is introduced and estimated using the data to avoid overfitting when *K* is too large (e.g. as *λ* → ∞, *f*(*x*) becomes linear) [12].

Regression coefficients ***β*** are estimated using restricted maximum likelihood, which imposes a smoothing penalty on the coefficients of the form

2.4 $$\lambda \beta^{T}P\beta \;,$$

where *λ* is the penalty parameter introduced earlier and *P* is a penalty matrix computed prior to model fitting (based on the type of smoothing spline chosen) [11,12]. The penalty parameter, matrix and basis functions can be estimated efficiently using the mgcv package [15]. Although the mgcv package uses empirical methods to estimate the parameters defining smoothing splines, the results can be interpreted from a Bayesian perspective.

### 2.3. Bayesian interpretation of penalized smoothing splines

The assumption that smoothing functions *f* are more smooth than wiggly can be considered a prior belief on the values that the coefficients can take. This prior can be formalized and incorporated into Bayesian inference by assuming the regression coefficients *β* have the prior distribution

2.5 $$\beta ∼N\left(0,\frac{P^{-}}{\lambda }\right),$$

where *P*^−^/*λ* is the covariance matrix [11,12]. However, the precision matrix *Pλ* is rank-deficient so is instead replaced by *P*_0_*λ*_0_ + *P*_1_*λ*_1,_ where the first term relates to a penalty on the null space of the smooth function and the second is the wiggliness penalty [16]. The interpretation of this is that the penalty matrix is separated into penalized components through *P*_1_ (relating to wiggly behaviour) and non-penalized components through *P*_0_. The splines *b_j_*(*x*) and penalty matrices can be efficiently generated using the jagam function in the mgcv package [16]. The definition of smoothing splines as linear combinations of (known) basis functions and (unknown) coefficients means that they can be entered into hierarchical models [17] and implemented using Bayesian inferential methods such as Markov chain Monte Carlo (MCMC). Under these conditions, the resulting penalized smoothing splines can be interpreted as random effects [11,18].

### 2.4. Spatial smoothing splines within Bayesian hierarchical models

In this study, we applied penalized smoothing splines to coordinates describing the relative ‘connectivity' of regions (e.g. coordinates of the centroid of regions). This created two-dimensional smooth surfaces describing spatial patterns in the data. Thin plate regression splines are relatively efficient at estimating smooths over multiple variables and do not require a surface to be stationary. In addition, thin plate regression splines have low posterior correlation between parameters, which improves mixing when using MCMC methods [19,20]. If a coordinate system does not currently exist that describes the connectivity in question, this can be created from a symmetric continuous measure using multi-dimensional scaling (MDS). MDS translates a continuous measure of ‘distance' or connectivity between observations onto an abstract Cartesian space and returns a set of coordinates [21]. For example, when connectivity is assumed to arise due to human movement, this could be defined as a continuous measure such as the number of air travel passengers, or an estimate from a movement model, such as a gravity or a radiation model [22,23], which assumes the number of people moving between areas is a function of population and distance. Note that MDS requires the measure of connectivity to be symmetric, for example, the number of people travelling to an area is assumed to be equal to the number returning.

Smooth surfaces were defined using splines and included in Bayesian hierarchical models of count data using the procedures detailed above. Models were implemented using NIMBLE [24,25], a flexible program that implements Bayesian models created in the BUGS language using MCMC methods within R [26]. The flexibility of this framework means that multiple spatially smooth surfaces can be included in the same model with different connectivity assumptions (e.g. distance-based and human movement). Interpreting the smooth surfaces over the various connectivity measures as random means the relative contribution of each spatial structure can be quantified by calculating the proportion of the overall variance of the random terms that is captured by each spatial term.

## 3. Simulation study 1: a single source of spatial structure

In this section, we present a simulation study in which we apply Bayesian spatial models to data generated from a distance-based spatial structure. We compare model performance between the penalized regression spline approach and a neighbourhood-based CAR model. A further simulation study assuming a single source of human movement-based connectivity is presented in the electronic supplementary material.

### 3.1. Data generation

Fictitious disease count data were generated from a Poisson distribution for each of the 1013 municipalities in South Brazil, the region used in the case study (§5), from model (2.1). The log of the population divided by 100 000, log(*ξ_i_*), was included as an offset (electronic supplementary material, figure S1). The population of each municipality was taken from the Brazilian census and described in §5.1. The intercept term *α* was set to zero, while the term *S_i_* was defined by

**Figure 1.**
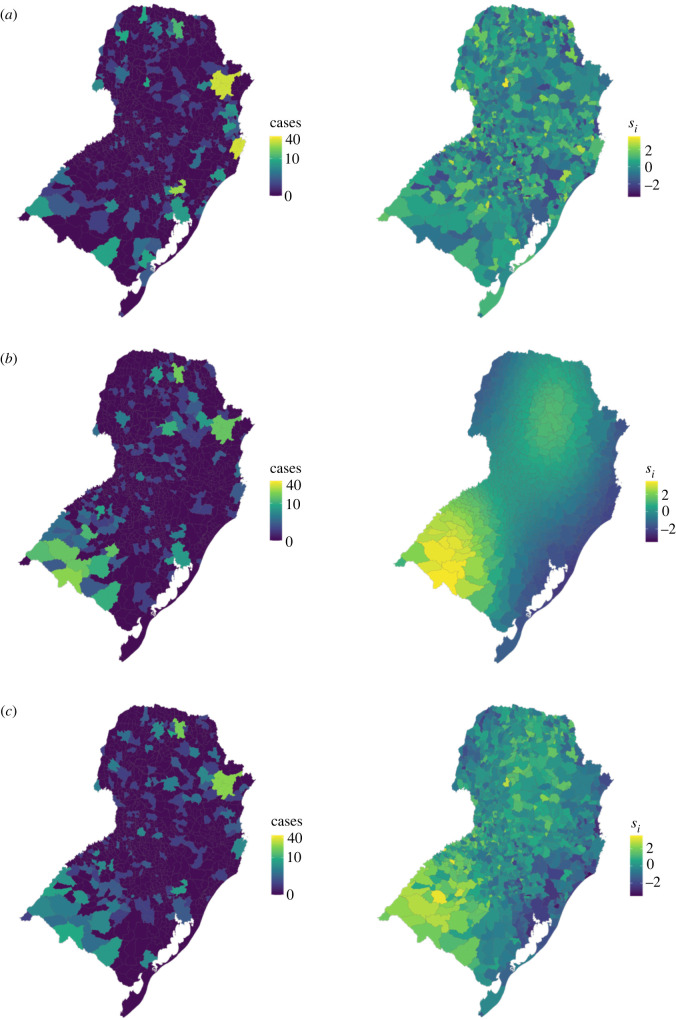
Simulated disease counts (left) and spatial random effects (right) under a distance-based structure using different spatial structure combinations. The number of cases simulated from a Poisson model and the underlying spatial structure where the data has (*a*) no spatial structure (*ϕ* = 0), (*b*) a distance-based structure only (*ϕ* = 1) and (*c*) equal contribution of both structures (*ϕ* = 0.5).

3.1 $$S_{i}=\sqrt{\phi }\cdot sm(x_{i},z_{i})+\sqrt{\left(1-\phi \right)}\cdot \varepsilon_{i},$$

where *ϕ* is a mixing parameter, taking values between 0 and 1, which measures the contribution of each term (if we interpret *sm*(*x_i_*, *z_i_*) as random and independent of ɛ*_i_*) to the overall variance of *S_i_*, and $\varepsilon_{i}∼N(0,1)$. *sm*(*x_i_*, *z_i_*) is a continuous function applied to connectivity coordinates (*x_i_*, *z_i_*) to emulate a spatially structured surface (figure 1*b*, taken from [27]):

3.2 $$\begin{array}{rl} sm(x,z) & =\pi \sigma_{x}\sigma_{z}(1.2e^{-{\left(x-0.2\right)}^{2}/\sigma_{x}^{2}-{\left(z-0.3\right)}^{2}/\sigma_{z}^{2}}\\ & \;+\;0.8e^{-{\left(x-0.7\right)}^{2}/\sigma_{x}^{2}-{\left(z-0.8\right)}^{2}/\sigma_{z}^{2}}) \end{array}$$

and

$$\sigma_{x}=0.3,\;\;\sigma_{z}=0.4.$$

To create a distance-based spatial structure, the smooth function *sm* was applied to coordinates of the centroid of municipalities which were scaled to take values between 0 and 1. The function *sm*(*x_i_*, *z_i_*) was centred at 0 by subtracting the overall mean from each value. Eleven simulated datasets were produced using equation (3.1), setting values of *ϕ* between 0 and 1 at intervals of 0.1 (figure 1).

### 3.2. Modelling approach

Two Poisson models containing spatially structured and unstructured random components were applied to each simulated dataset

$$y_{i}∼\mathrm{Poisson}(E(y_{i}))$$

3.3 $$\log(E(y_{i}))=\log(\xi_{i})+\alpha +u_{i}+v_{i}$$

3.4 $$\log(E(y_{i}))=\log(\xi_{i})+\alpha +\frac{1}{\tau }\left(\sqrt{\phi }\cdot u_{*i}+\sqrt{1-\phi }\cdot v_{*i}\right)\;.$$

In model (3.3), *u_i_* is a spatially structured term, constructed using a thin plate regression spline on the coordinates of the centroid of each municipality, and *v_i_* is a spatially unstructured term, assumed to follow a zero-mean normal distribution, representing heterogeneity between regions. This spatially smooth model was compared with a more conventional random effect approach based on the BYM2 model (model (3.4)), which is often used to capture spatial structure in disease mapping [3,28,29]. In model (3.4), u_*__i_ are spatially structured random effects assuming a CAR model with a binary neighbourhood matrix (see equation (2.2)), v_*__i_ are unstructured normal random effects, and *ϕ* is a mixing parameter, measuring the contribution of each random effect to the marginal variance $(1/\tau^{2})\;$of the overall random effect [3,28]. Here, *ϕ* = 1 represents a purely spatial model, equivalent to an intrinsic CAR model [30], and *ϕ* = 0 indicates no spatial structure in the data. Spatially smooth models were fitted using MCMC simulations in R via the NIMBLE package [24]. Although the BYM2 model can be formulated and fitted using MCMC simulations [31], we found that most contemporary disease mapping studies use integrated nested Laplace approximations (INLA) for model fitting [32]. INLA is an approximate Bayesian inference approach which provides a more efficient alternative to MCMC and avoids issues with convergence [14,29]. We compared the spatially smooth model with a BYM2 model fitted using INLA to ensure we were comparing our results to the conventional approach. However, to ensure any differences were not a result of inferential methods, the BYM2 random effect model was also fitted using MCMC simulations in NIMBLE and compared with the spatially smooth model. Results of this comparison are presented in the electronic supplementary material.

Model comparison was based on mean absolute error (MAE) and Watanabe–Akaike information criterion (WAIC), an information criterion used to assess the predictive accuracy of Bayesian models [33]. Lower values of MAE and WAIC are preferred. The relative contribution of the spatially structured term, *u_i_*, to the overall random terms in the spatially smooth model was defined as the proportion of the overall random term variance explained by *u* (var(*u*)/var(*u* + *v*)). This was estimated using samples from the posterior distribution of *u* and *v*. We compared estimates of the *ϕ* hyperparameter from INLA, the relative contribution of *u_i_* with the random effect variance from NIMBLE, and the known proportion of spatial variance used in the simulation. All analyses were carried out using R v. 4.1.1 [26]. The code used to simulate data and perform analyses is available here: https://doi.org/10.5281/zenodo.7054457 [34].

### 3.3. Results

**Figure 2.**
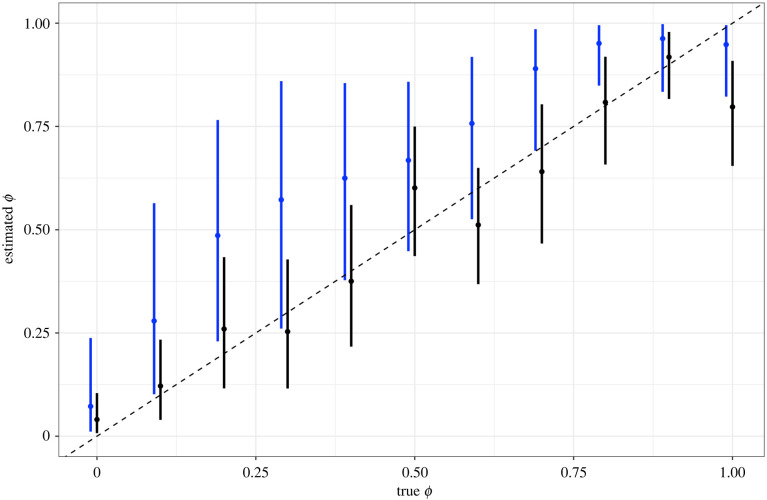
The mean and 95% credible interval of estimated *ϕ* values extracted from models including a smoothing spline (black) and BYM2 (blue) compared with the known value (dashed line). Estimated *ϕ* values for the smoothing spline model were calculated using the proportion of the random effect variance explained by the spatially structured term and were extracted from INLA output for the BYM2 model.

We found that the spatial spline model estimates were closer to the true value of *ϕ* than the BYM2 model for most simulations (figure 2 and table 1), and that INLA's estimates of this parameter were not always consistent with the true value. This indicates that the spatial spline models were able to identify and quantify the relative contribution of this spatial structure within the data as well as (if not better than) INLA's BYM2 models.

**Table 1.** Model comparison statistics and mean estimates of the mixing parameter, *ϕ*, from the smoothing spline and INLA BYM2 models. Mean absolute error (MAE) and WAIC calculated for the spatial spline and BYM2 models for each simulated dataset. The lowest MAE and WAIC, and the *ϕ* estimate closest to the value used in each simulation are highlighted in italics.

*ϕ*	smoothing spline model			INLA BYM2 model		
	MAE	WAIC	*ϕ* estimate	MAE	WAIC	*ϕ* estimate
0	1.51	*996.94*	*0.041*	*1.04*	1005.79	0.072
0.1	1.54	*1030.64*	*0.121*	*1.11*	1034.29	0.279
0.2	1.33	932.42	*0.26*	*1.04*	*931.79*	0.486
0.3	1.27	*909.42*	*0.253*	*0.93*	912.5	0.572
0.4	1.39	*961.67*	*0.375*	*1.08*	976.12	0.625
0.5	1.54	*935.09*	*0.601*	*1.21*	954.34	0.668
0.6	1.5	*881.09*	*0.512*	*1.13*	973.61	0.757
0.7	1.45	*931.85*	*0.641*	*1.17*	989.24	0.89
0.8	1.63	*947.51*	*0.808*	*1.37*	983.96	0.951
0.9	1.59	*876.37*	*0.918*	*1.37*	922.29	0.963
1	1.48	*875.42*	0.797	*1.25*	924.14	*0.948*

MAEs and WAIC values show that model performance was similar between the smoothing spline and BYM2 models (table 1). The WAIC showed the smoothing spline model performed slightly better on all simulated datasets apart from one, although the MAE preferred the BYM2 models. When these approaches were compared with the BYM2 model fitted using MCMC (electronic supplementary material, S1), we found that some of these differences appear to be a result of fitting the model using INLA rather than model formulation itself. However, the objective of this comparison was not to show that the proposed smooth model outperforms these approaches, rather that it performs as well as the current standard. These results illustrate that the smoothing spline was able to detect spatial connectivity between neighbouring regions while being flexible enough to capture alternative structures. The 95% credible interval (CI) of the intercept coefficient estimate contained the true value 0 for all models for both approaches (electronic supplementary material, figure S2).

## 4. Simulation study 2: two sources of spatial structure

In this section, we present another simulation study in which we apply Bayesian spatial models to data generated with two sources of spatial connectivity: distance-based and human movement-based.

### 4.1. Data generation

An extension of the spatial term, *S_i_*, in equation (3.1) was used to generate data with spatial connectivity arising from two different sources

4.1 $$S_{i}=\sqrt{\phi_{1}}\cdot sm(a_{i},b_{i})+\sqrt{\phi_{2}}\cdot sm(c_{i},d_{i})+\sqrt{\phi_{3}}\cdot \varepsilon_{i}$$

and

$$\phi_{1}+\;\phi_{2}+\;\phi_{3}=1.$$

Where *sm* is a smooth function (equation (3.2)), applied to coordinates describing distance-based connectivity (*a_i_*, *b_i_*), and human movement-based connectivity (*c_i_*, *d_i_*). The coordinates of the centroid of municipalities were scaled to take values between 0 and 1 and used to describe distance-based connectivity (*a_i_*, *b_i_*). As a coordinate system describing connectivity arising from human movement does not exist, we applied MDS to an estimate of the number of people moving between municipalities, generated using a movement model described in the electronic supplementary material, to create coordinates *c_i_* and *d_i_* (electronic supplementary material, figure S3).

**Figure 3.**
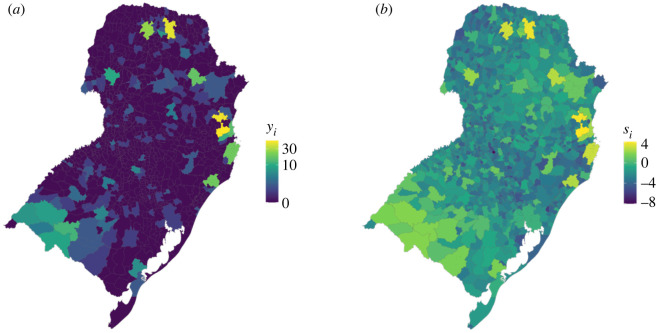
Simulated data containing two sources of spatial structure. Simulated disease counts, *y_i_* (*a*) and spatial random terms, *s_i_* (*b*), for South Brazil generated using equation (4.1), where *ϕ*_1_ = 0.4, *ϕ*_2_ = 0.5 and *ϕ*_3_ = 0.1.

In this example, we used three scaling parameters, *ϕ*_1_, *ϕ*_2_ and *ϕ*_3_, to describe the relative contribution of each random term to the marginal variance. We held *ϕ*_3_ constant at 0.1, with *ϕ*_1_ and *ϕ*_2_ taking values between 0 and 0.9 at intervals of 0.1, creating 10 simulated datasets (figure 3).

### 4.2. Modelling approach

We applied a Poisson spatial model to each simulated dataset which contained three random terms

$$y_{i}∼\mathrm{Poisson}(E(y_{i}))$$

4.2 $$\log(E(y_{i}))=\log(\xi_{i})+\alpha +u_{1,i}+u_{2,i}+v_{i}.$$

Where *u*_1,*i*_ is constructed using a thin plate regression spline applied to coordinates of the centroids of municipalities, and *u*_2,*i*_ is structured using a thin plate regression spline applied to human movement-based connectivity coordinates described previously. *v_i_* is assumed to have no spatial structure and represents unobserved heterogeneity between municipalities. We compared the proportion of the marginal variance explained by each random term and compared these with the known *ϕ* values used in data generation.

### 4.3. Results

**Figure 4.**
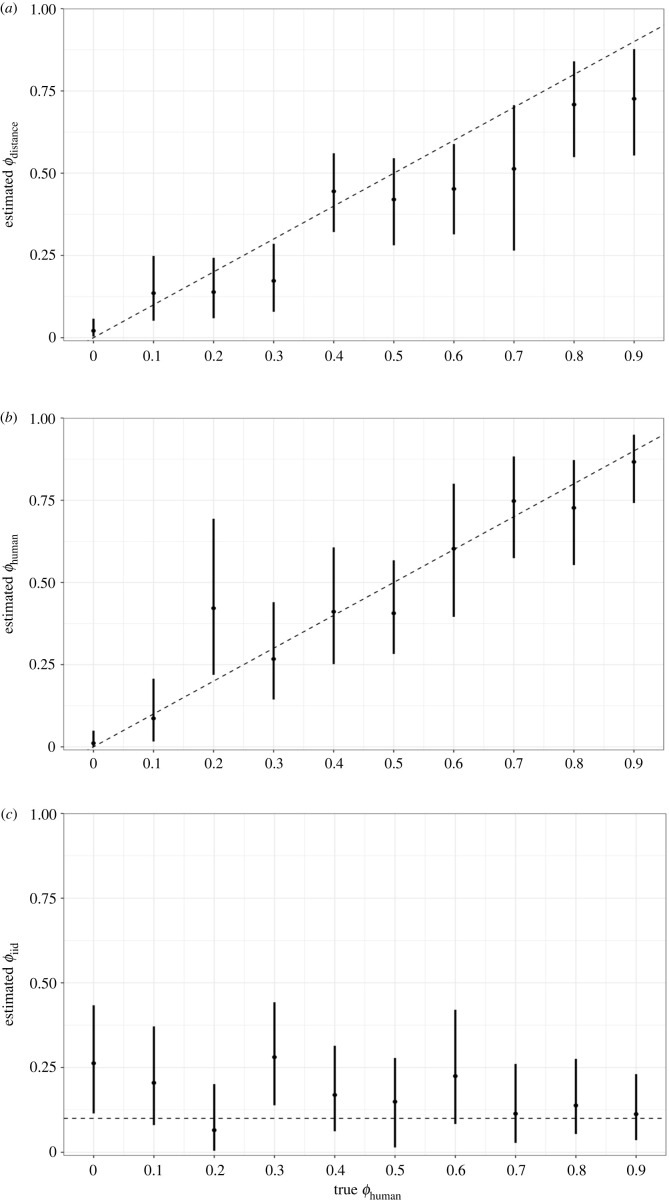
Mean and 95% credible interval of the proportion of variance of the random effects explained by (*a*) the distance-based structured term, (*b*) the human movement-based structured term and (*c*) unstructured random term. Dashed lines represent the true value from simulations.

We found that the models were able to accurately estimate the intercept coefficient value of 0 across most simulated datasets (electronic supplementary material, figure S4). Estimates of the relative contribution of each random term to the overall spatial structure were close to *ϕ* values used in simulations and were able to detect the increasing contributions of distance-based and human movement-based terms as the true value increased (figure 4).

## 5. Case study

This case study uses the Bayesian spatially smooth models introduced in previous sections to map the spatial patterns of dengue incidence in South Brazil between 2001 and 2020.

### 5.1. Data description

**Figure 5.**
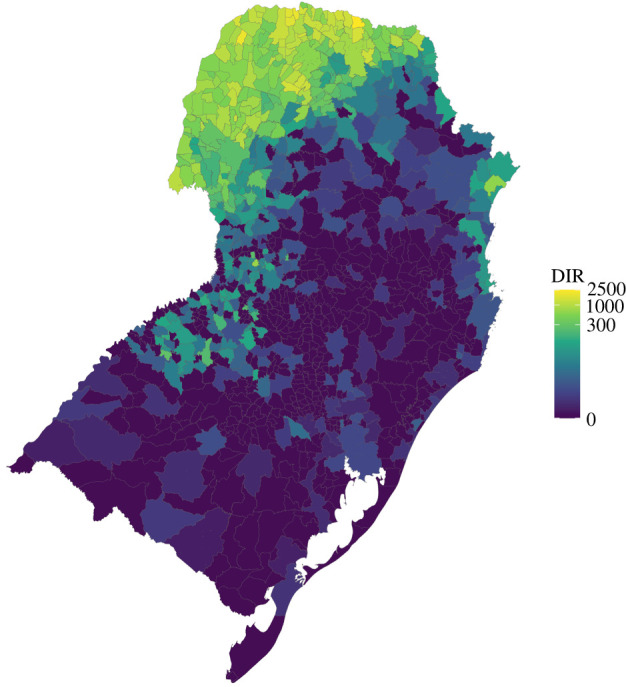
Average dengue incidence rate (DIR), 2001–2020 in South Brazil. The mean annual dengue incidence rate per 100 000 residents in South Brazil from 2001 to 2020. Data are shown on a log scale.

We obtained annual notified dengue cases for each of South Brazil's 1013 municipalities between 2001 and 2020 from Brazil's Notifiable Diseases Information System, freely available via the Health Information Department, DATASUS (https://datasus.saude.gov.br/informacoes-de-saude-tabnet/). To explore the pattern of disease over the whole period, we took the average annual number of cases over the period and rounded this to the nearest whole number. The annual population for each municipality was obtained from the Brazilian Institute of Statistics and Geography (IBGE) via DATASUS (http://tabnet.datasus.gov.br/cgi/deftohtm.exe?ibge/cnv/poptbr.def) over the same period and aggregated in the same way. We used the population divided by 100 000 as an offset to model the dengue incidence rate (DIR), a measure used by the Brazilian Ministry of Health to monitor dengue outbreaks. South Brazil was previously thought to be protected from dengue due to its temperate climate, with winter temperatures too low for the primary vector, *Aedes aegypti*, to breed and transmit the disease. However, recent studies have shown that the northern part of the South region now experiences outbreaks, thought to be due to increasing temperatures (figure 5, [35]). The data show a clear distance-based spatial pattern in this region. However, studies of other temperate regions of South America, such as Argentina, have hypothesized that increased outbreaks in cooler regions may be a result of human movement into previously protected cities [7,36]. Data used in this case study are available from https://doi.org/10.5281/zenodo.7054457 [34].

### 5.2. Modelling approach

We applied a negative binomial model to the average annual dengue cases, using the log of the population divided by 100 000 as an offset to explore the DIR in South Brazil. A negative binomial distribution was assumed to account for possible overdispersion in the dengue case count [5]. Model (4.2) was applied to the data, spatial random terms were structured by applying thin plate regression splines to the coordinates of the centroids of municipalities (*u*_1,*i*_, assuming distance-based connectivity), and human movement-based connectivity coordinates described in §4 and the electronic supplementary material (*u*_2,*i*_).

### 5.3. Results

**Figure 6.**
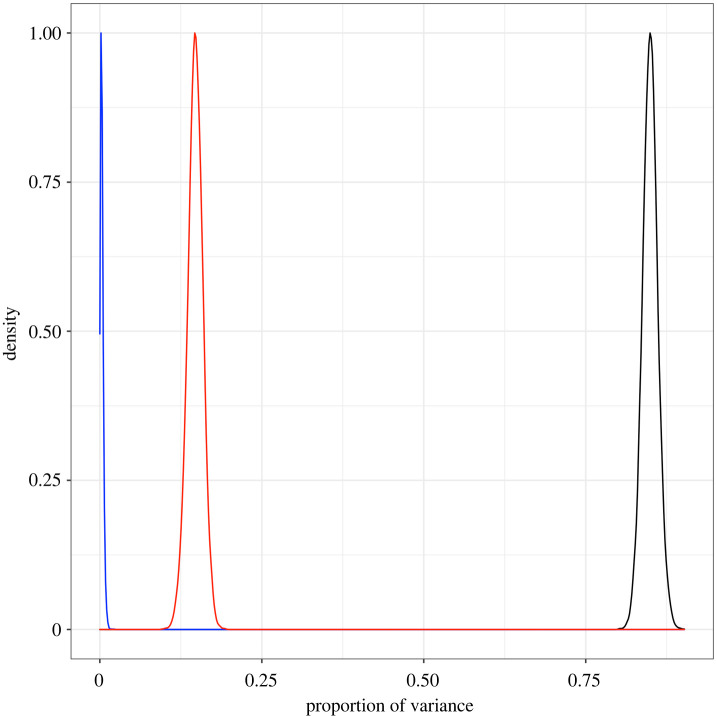
Estimates of the proportion of variance explained by distance-based (black), human movement-based (blue) and independent (red) random terms. Using simulations extracted from NIMBLE, the variance of each random term was calculated and divided by the variance of the combined random component, giving the relative contribution of each structure.

The model found that human movement did not account for much of the spatial structure of the data in this region (*ϕ*_2_ = 0.003, 95% CI: 0, 0.012), and most of the variation could be attributed to the distance-based random term (*ϕ*_1_ =0.85, 95% CI: 0.823, 0.876, figure 6). The human movement data used to create these random effects were only able to capture movement between cities in South Brazil. However, outbreaks in temperate regions such as this are likely to be triggered by the movement of people from endemic regions elsewhere in Brazil into the South [7].

**Figure 7.**
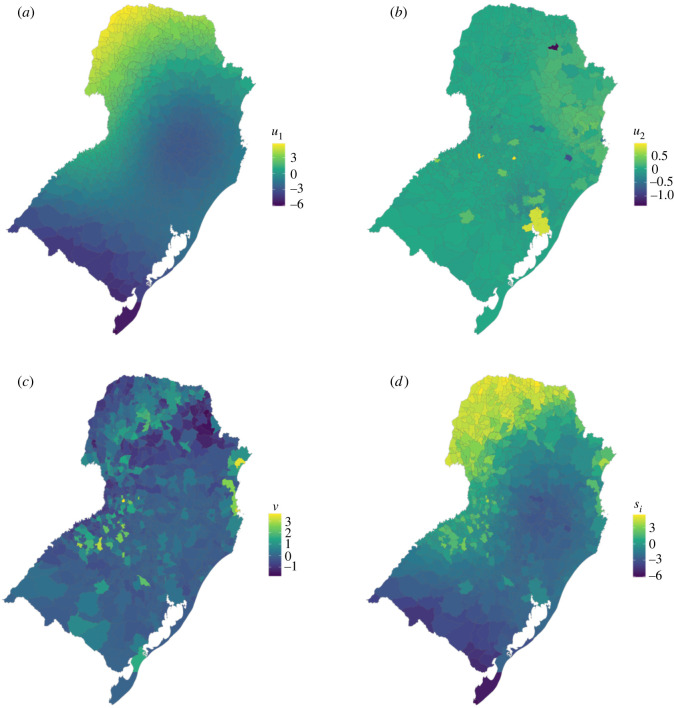
Mean estimates of the (*a*) distance-based, (*b*) human movement-based, (*c*) unstructured and (*d*) combined random terms.

Estimates of each random term and the combined total were extracted and plotted to generate hypotheses about these patterns (figure 7). Most of the spatial structure came from the distance-based random term, which shows the highest risk was in the northwest and that the risk decreased to the south. This area of increased risk is the same region which was found to have an increase in the number of months per year with temperatures suitable for dengue transmission since 2010 in a previous study [35]. This model could be extended to include temperature and other variables known to influence dengue risk.

## 6. Discussion

In this paper, we have shown that penalized smoothing splines present a flexible alternative to CAR-based structures of spatial random effects that allow multiple sources of spatial connectivity to be considered within the same model. Smoothing splines allow the spatial structure to be derived from data as part of the model fitting process, producing a non-stationary spatial surface specific to the data being considered. This smooth surface can be extracted and plotted to generate hypotheses about the reasons for this spatial connectivity which may help identify potential drivers of disease. Although many disease mapping studies assume a distance-based structure of connectivity, the smooth spline approach used here can be applied to any symmetric continuous measure of connectivity, including human movement. Another benefit of the smoothing spline approach is that the model structure can be extended to include multiple sources of spatial connectivity and can produce parameters quantifying the relative contribution of each structure to the underlying variance of the data. Although this study has focused on disease mapping models of count data, we have shown this method is compatible with other models, such as logistic models for binary data (see the electronic supplementary material).

Formulating models in NIMBLE (or other similar coding languages) and implementing them using MCMC methods allows for flexibility and complexity in the model structure. However, these models are more likely to face issues with convergence than approximate methods such as INLA [14]. MCMC methods may also take longer than INLA to fit models if convergence is an issue, although this is not always the case when using NIMBLE [37].

One of the main benefits of using penalized smoothing splines over CAR-based priors is that they can be applied to any symmetric continuous measure of connectivity. However, the most appropriate measure may not always be clear or available. For example, human movement-based connectivity can be captured using data to describe regular, short-distant movement such as commuting within a city, or long-distance, long-term movement such as migration, which requires different assumptions [9]. Mobile phone data have potential to describe short-term movements at small spatial scales but may be difficult to obtain, and care must be taken in some settings where bias may arise [38]. Movement models, such as gravity and radiation models, assume that the number of people moving between areas can be described as a function of population and distance [22]. Movement models provide an alternative when data is unavailable or inappropriate and have been shown to replicate patterns of movement in large cities and European countries [23,39]. However, care must be taken when parametrizing these models, particularly in rural settings [40]. Although distance is recognized as an important driver of human movement [22], our simulation studies showed that this approach can distinguish between the relative contribution of both sources of connectivity to the overall spatial structure (see §4 and electronic supplementary material, S4).

One limitation of this method is that the measure of connectivity must be symmetric to produce a spatially smooth surface. This is often not realistic when considering human movement, as the number of people moving from smaller to larger cities is often different to those moving in the opposite direction [41]. In the examples presented in this study, we assumed that the number of people travelling between municipalities is equal to the number of people returning. Also, the models presented in this study only consider a single time point (or data summarized over a given time period); however, disease risk is likely to vary over time and models may be required to account for inter-annual or seasonal variation. Data presented in the South Brazil study have been used elsewhere to show the expansion of dengue outbreaks into the region and the changes in spatial structure over the past 20 years [35,42]. The models presented here can be extended to include temporal covariates or random terms to account for seasonal and annual trends, and changing spatial connectivity surfaces to reflect changing patterns of movement. Tensor smooth functions, a type of smoothing spline which allows interaction between variables measured on different scales [27], may be incorporated to explore the interactions between time and connectivity. These structures can be explored to understand changing dynamics of diseases and generate hypotheses about drivers of change or highlight areas at risk. Covariates such as climate indicators can also be included into the models and random term estimates compared to highlight the relative variability in the disease risk explained by these covariates.

Penalized smoothing splines present a flexible alternative to conventional random effect structures when constructing Bayesian hierarchical models. They require minimal user assumptions beyond smoothness and can be applied to any symmetric continuous measure of connectivity. By taking a Bayesian view of these smoothing splines, we can incorporate multiple sources of spatial connectivity into a complex modelling framework efficiently and quantify their relative contribution to the overall spatial structure of the data. This is particularly useful in infectious disease epidemiology where the drivers of transmission may be complicated and not fully understood.

## Data Availability

All data used in this study are open access and available freely on the internet; see the methods section for more details. Data and code used to produce this analysis is available from Zenodo (https://doi.org/10.5281/zenodo.7054457) [34]. The data are provided in the electronic supplementary material [43].
